# Successful use of nemolizumab for refractory pruritus in cutaneous T-cell lymphoma

**DOI:** 10.1016/j.jdcr.2026.07.012

**Published:** 2026-07-28

**Authors:** Christine Li, Julia Rogers, Haya Raef, Laura Aspey

**Affiliations:** a University of Massachusetts Chan Medical School, Worcester, Massachusetts; b Emory University School of Medicine, Atlanta, Georgia; c Department of Dermatology, Emory University School of Medicine, Atlanta, Georgia; d Department of Dermatology, Grady Memorial Hospital, Atlanta, Georgia

**Keywords:** cutaneous T cell lymphoma, IL-31, mycosis fungoides, nemolizumab, pruritus, Sezary syndrome

## Introduction

Cutaneous T-cell lymphoma (CTCL) encompasses a group of non-Hodgkin lymphomas characterized by malignant proliferation of skin-homing T cells.^1^ Mycosis fungoides (MF) is the most common subtype and often presents with patches, plaques, and tumors that can cause intense and chronic pruritus.^2^ Pruritus in CTCL can be severe and refractory to standard therapies, significantly impairing patients’ quality of life, sleep, and mental health.^2^^,^^3^ The mechanism of pruritus in CTCL is multifactorial, involving numerous cytokines, chemokines, neuropeptides, and other inflammatory mediators.^2^, ^3^, ^4^ Interleukin-31 (IL-31), a cytokine produced by activated T cells, has been identified as a key mediator of pruritus in inflammatory dermatoses, including prurigo nodularis and atopic dermatitis.^2^ IL-31 exerts its effects by binding to IL-31 receptor A (IL-31RA), expressed on sensory neurons and keratinocytes, leading to neural sensitization and itch transmission.^4^ Nemolizumab, a humanized monoclonal antibody targeting IL-31RA, is approved by the US Food and Drug Administration for prurigo nodularis and moderate-to-severe atopic dermatitis.^5^ To date, there have only been two documented cases describing nemolizumab use for CTCL-associated pruritus.^6^ Here, we report a case of a 54-year-old male with erythrodermic MF who presented with refractory pruritus that was successfully managed with nemolizumab.

## Case report

A 54-year-old African American male with a history of Stage IVA2 (T4N3M0B0) erythrodermic MF on palliative gemcitabine chemotherapy was admitted to the hospital with fever, nausea, myalgias, and painful draining wounds in the bilateral axillae. Blood and skin cultures were positive for methicillin-resistant *Staphylococcus aureus*, supporting a secondary infectious complication of advanced MF. Dermatology was consulted for management of refractory pruritus.

One year prior, the patient presented to the dermatology clinic with erythroderma of unclear etiology. A punch biopsy of the left arm showed vacuolar interface dermatitis and an associated lymphocytic infiltrate with intraepidermal lymphocytes. MF was diagnosed based on clinical presentation, biopsy results, and the detection of clonal T-cell receptor gamma gene rearrangements in both skin and blood samples. Prior to the current admission, the patient’s MF was treated with topical corticosteroids, narrowband UVB therapy, and acitretin, followed by methotrexate and bexarotene. At the time of admission, his treatment regimen included total skin radiation therapy and palliative gemcitabine chemotherapy guided by oncology.

Upon admission, the patient reported severe pruritus that remained poorly controlled despite treatment with aprepitant, diphenhydramine, famotidine, doxepin, and a prednisone taper. Physical examination was notable for pustules on the scalp, face, and neck; macerated skin in the axillae; and diffuse skin erythema, desquamation, and lichenification (Fig 1). He was started on triamcinolone ointment and intravenous daptomycin for superimposed superficial skin infection and continued on his home regimen for pruritus. Upon discharge from the hospital, he was initiated on nemolizumab with a 60 mg loading dose followed by 30 mg maintenance dose every 28 days. The patient reported complete resolution of pruritus 7 days after a single dose of nemolizumab, with sustained improvement 179 days later, and denied any associated adverse effects.

**Fig 1:**
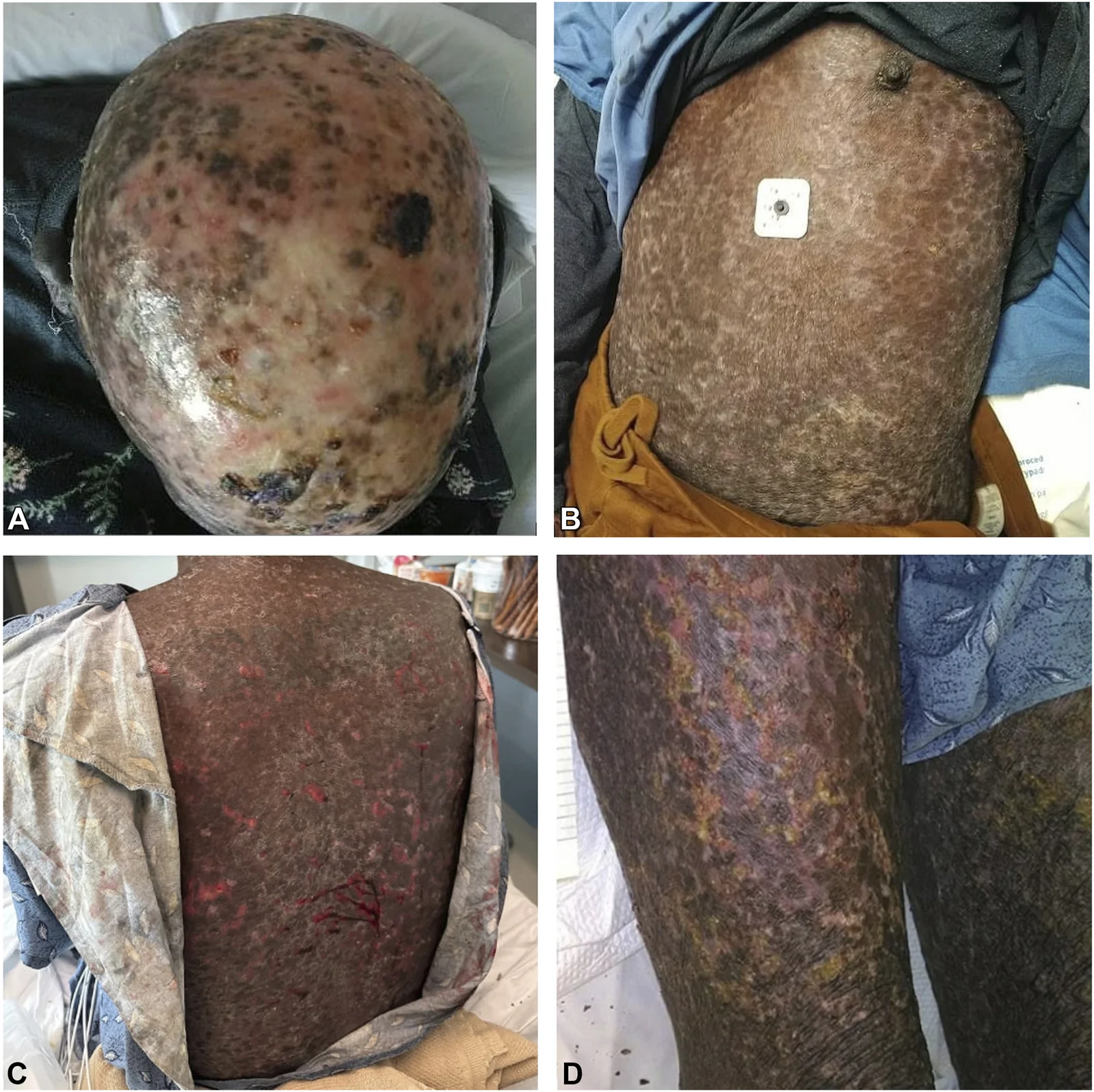
Clinical presentation prior to initiating nemolizumab: Diffuse erythema involving >90% of total body surface area, including the **(A)** scalp, **(B)** chest, **(C)** back, and **(D)** upper and lower extremities, with prominent lichenification, desquamation, excoriations, and alopecia.

## Discussion

Chronic pruritus affects up to 94% of patients with CTCL and is frequently reported as the most bothersome symptom affecting quality of life.^7^ CTCL-associated pruritus can be generalized or restricted to lesional skin, and it is typically worse in late-stage disease.^3^ Treatment of pruritus is a crucial component of CTCL management, although this is challenging as patients often experience limited or transient relief from conventional itch therapies.^3^ Management of CTCL-associated pruritus may focus on targeting the lymphoma or the pruritus. Lymphoma-targeted therapies, including skin-directed therapies (eg, topical corticosteroids, topical chemotherapy agents, phototherapy) and systemic agents (eg, vorinostat, alemtuzumab), may decrease pruritus by reducing cutaneous disease.^3^ Itch-targeted therapies, including aprepitant, naloxone, naltrexone, butorphanol, mirtazapine, gabapentin, and thalidomide, have been employed with variable success in case reports.^3^ Despite these options, CTCL-associated pruritus remains difficult to manage, underscoring the need for additional therapies that directly target the underlying pathways involved.

The pathogenesis of pruritus in CTCL is complex and not fully elucidated, but cytokines including IL-4, IL-5, IL-10, IL-13, IL-25, and IL-31 appear to play important roles.^2^, ^3^, ^4^ IL-31, produced by activated T cells, has been posited to play a central role in CTCL-associated pruritus and induces itch by stimulating IL-31RA on sensory neurons and keratinocytes.^2^ In CTCL, increased serum IL-31 levels and Th2-skewed cytokine profiles have been reported, providing a rationale for IL-31 pathway inhibition in this setting.^8^ Furthermore, several studies have demonstrated that serum IL-31 levels correlate with disease severity and pruritus intensity in CTCL, serving as a potentially useful tool for disease monitoring during treatment.^8^^,^^9^

Nemolizumab is a subcutaneously administered humanized monoclonal antibody that selectively targets IL-31RA and is approved in the United States for atopic dermatitis and prurigo nodularis. Nemolizumab has demonstrated significant efficacy with rapid reductions in itch severity and sleep disturbance, while maintaining a favorable safety profile.^5^ The demonstrated ability of nemolizumab to suppress severe, treatment-refractory pruritus quickly and durably in these other primarily Th2-driven inflammatory dermatoses provides a strong mechanistic and clinical rationale for its use in CTCL-associated pruritus.

To date, evidence on the efficacy of IL-31 blockade for malignancy-associated pruritus remains sparse. Only two cases have been documented of CTCL-associated pruritus successfully treated with nemolizumab, marking our case as the third (Table I). Our patient experienced relief of severe, refractory pruritus after receiving one dose of nemolizumab, with marked improvement in sleep and daily functioning. Of note, the patient was also on total skin radiation therapy and gemcitabine chemotherapy, so the improvement in pruritus may have been partially attributable to response of the underlying lymphoma to these therapies. Nevertheless, the temporal association between nemolizumab administration and rapid symptomatic improvement suggests that IL-31 receptor blockade contributed meaningfully to pruritus control. Importantly, there was no evidence of CTCL progression over 9 months of follow-up (Fig 2), suggesting that IL-31 receptor antagonism did not exacerbate disease activity. However, the use of biologic agents in CTCL warrants caution given the risk of immune modulation and uncertain long-term effects on malignant T-cell populations. Although no adverse effects or laboratory abnormalities were observed in this case, formal studies with longer follow-up are needed.

**Table I:** Clinical presentation, medication history for pruritus management, and response to nemolizumab for patients with cutaneous T-cell lymphoma-associated pruritus∗

Clinical presentation				Medications tried and failed for pruritus			Response to nemolizumab			Reference
Pt.	Sex	Age	Relevant history (including comorbidities that predispose to pruritus)	Topical therapies or light treatment	Antihistamines or neuropathic medications	Systemic antibiotics or anti-inflammatory medications	Improvement in pruritus	Adverse effects on nemolizumab	Concomitant therapies while on nemolizumab	
1	F	63 y.o.	24-y history of mycosis fungoides Past medical history of renal insufficiency and prediabetes	Desonide ointment, fluocinonide ointment, triamcinolone ointment, fluticasone cream, mometasone ointment, clobetasol ointment, clotrimazole-betamethasone cream, alclometasone ointment, ruxolitinib cream	Hydroxyzine, fexofenadine, diphenhydramine, loratadine, cetirizine, gabapentin	Multiple prednisone tapers, intramuscular triamcinolone, hydroxychloroquine, dupilumab (12 mo), omalizumab (18 mo), upadacitinib (8 mo), abrocitinib (2 mo), subcutaneous methotrexate (4 mo)	100% improvement at day 34, sustained improvement at day 149	None	Clobetasol ointment as needed until Day 114	Murase et al†
2	F	82 y.o.	7-y history of mycosis fungoides	Triamcinolone ointment, clobetasol ointment, betamethasone ointment, fluocinolone oil, hydrocortisone ointment	Doxepin, hydroxyzine, cetirizine, gabapentin, diphenhydramine	Multiple prednisone tapers, dupilumab	100% improvement at day 30	None	None	Murase et al†
3	M	54 y.o.	1-y history of Stage IVA2 (T4N3M0B) erythrodermic mycosis fungoides Past medical history of prediabetes	Triamcinolone ointment, narrowband UVB therapy (14 sessions)	Aprepitant, diphenhydramine, famotidine, doxepin, gabapentin	Prednisone taper, acitretin (4 mo), methotrexate (2 mo), bexarotene (2 mo)	100% improvement at day 7, sustained improvement at day 179	None	Ongoing use of triamcinolone ointment as needed	Our case

∗ All three patients received a 60 mg loading dose of nemolizumab followed by 30 mg maintenance dose every 28 days.

† References: Murase EM, Dulai AS, Marzouk S, Lio PA, Sivamani RK, Murase JE. Rapid clinical response to nemolizumab in dermatologic diseases associated with pruritus and burning: A multicenter case series. Journal of the American Academy of Dermatology. Aug 2025;93(2):466-468. https://doi.org/10.1016/j.jaad.2025.03.047.

**Fig 2:**
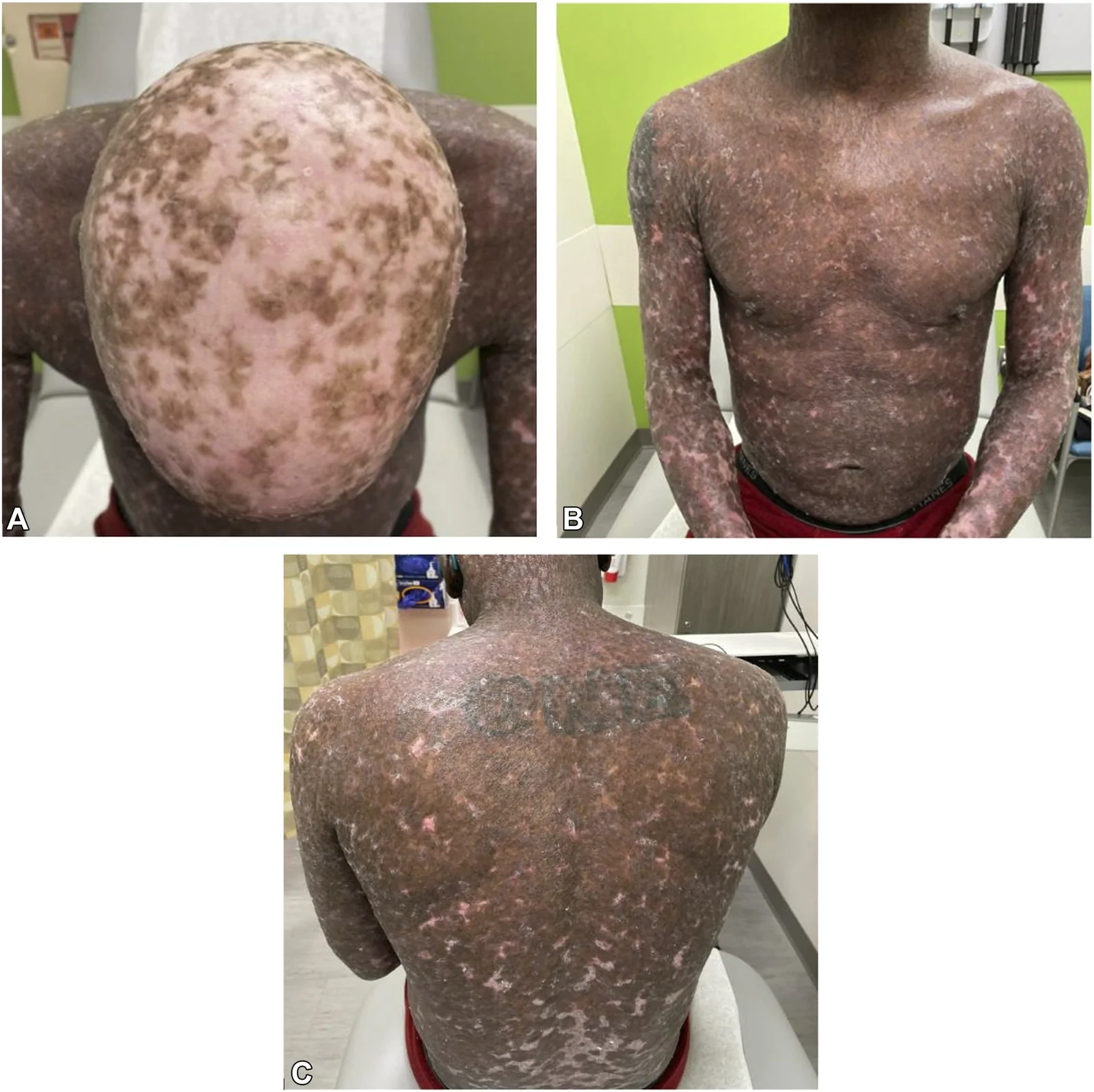
Clinical presentation 6 months after initiating nemolizumab, (**A-C**): In comparison to his presentation before initiating nemolizumab, the areas of erythema, excoriations, and lichenification appear improved. Notably, the patient was concurrently on total skin radiation therapy and gemcitabine chemotherapy, which may have also contributed to symptomatic improvement.

Our findings demonstrate that nemolizumab can provide rapid and sustained relief of pruritus in patients with CTCL, without significant adverse effects or apparent disease progression, although concurrent lymphoma-directed therapy may have also contributed to symptomatic improvement. IL-31 receptor blockade may represent a promising therapy for CTCL-associated pruritus. Controlled clinical studies are warranted in the future to further evaluate the efficacy and safety of nemolizumab use for CTCL-associated pruritus.

## Conflicts of interest

None disclosed.
